# Case mix-based changes in health status: A prospective study of elective surgery patients in Vancouver, Canada

**DOI:** 10.1177/13558196231182630

**Published:** 2023-06-11

**Authors:** Jason M Sutherland, R Trafford Crump, Ahmer A Karimuddin, Guiping Liu, Kevin Wing, Arif Janjua, Kathryn Isaac

**Affiliations:** 1 Centre for Health Services and Policy Research, School of Population and Public Health, 8166 University of British Columbia, Vancouver, Canada; 2 Department of Surgery, 2129 University of Calgary, Calgary, Alberta; 3 Section of Colorectal Surgery, Department of Surgery, 8166 University of British Columbia, Vancouver, Canada; 4 Centre for Health Services and Policy Research, 8166 University of British Columbia, Vancouver, Canada; 5 Department of Orthopaedics, 8166 University of British Columbia, Vancouver, Canada; 6 Rhinology and Skull Base Surgery, Department of Surgery, Division of Otolaryngology - Head and Neck Surgery, 8166 University of British Columbia, Vancouver, Canada; 7 Plastic Surgery, 8166 University of British Columbia, Vancouver, British Columbia, Canada

**Keywords:** case mix, patient-reported outcomes, planned surgery

## Abstract

**Introduction:**

Hospital activity is often measured using diagnosis-related groups, or case mix groups, but this information does not represent important aspects of patients’ health outcomes. This study reports on case mix-based changes in health status of elective (planned) surgery patients in Vancouver, Canada.

**Data and methods:**

We used a prospectively recruited cohort of consecutive patients scheduled for planned inpatient or outpatient surgery in six acute care hospitals in Vancouver. All participants completed the EQ-5D(5L) preoperatively and 6 months postoperatively, collected from October 2015 to September 2020 and linked with hospital discharge data. The main outcome was whether patients’ self-reported health status improved among different inpatient and outpatient case mix groups.

**Results:**

The study included 1665 participants with completed EQ-5D(5L) preoperatively and postoperatively, representing a 44.8% participation rate across eight inpatient and outpatient surgical case mix categories. All case mix categories were associated with a statistically significant gain in health status (*p* < .01 or lower) as measured by the utility value and visual analogue scale score. Foot and ankle surgery patients had the lowest preoperative health status (mean utility value: 0.6103), while bariatric surgery patients reported the largest improvements in health status (mean gain in utility value: 0.1515).

**Conclusions:**

This study provides evidence that it was feasible to compare patient-reported outcomes across case mix categories of surgical patients in a consistent manner across a system of hospitals in one province in Canada. Reporting changes in health status of operative case mix categories identifies characteristics of patients more likely to experience significant gains in health.

## Introduction

Many countries pay hospitals on the basis of activity, that is, the number and type of patients they treat.^1,2^ There is increasing recognition that this approach creates incentives for hospitals to maximise volume of care over corresponding measures of quality or effectiveness. Moving from volume- and case mix-based measures towards patient-centred outcome measures requires appropriate understanding of how patient’s health changes following a given intervention.

One such approach is through patient-reported outcome measures. Patient-reported outcomes (PROs) are collected through validated survey instruments (PROMs) where patients self-report their symptom severity, health status or health-related quality of life,^3,4^ thus enabling standardised measurement of patient outcomes. Originally developed to measure symptom change in clinical trials,^5,6^ PROs are increasingly used to measure intervention impact and effectiveness.^7–9^ Countries such as England have implemented routine collection of PROs to measure health gain in patients undergoing selected surgical procedures.^10^ and understand variation in hospital performance,^11^ There is increasing interest in the wider application of PROs for quality improvement^12^ and possible reimbursement purposes (‘value-based payment’).^13–15^

This study seeks to contribute to our understanding of the use and usefulness of PROs for measuring changes in patient’s health status across a range of elective (planned) surgery case mix categories in six hospitals in Vancouver, Canada. Exploratory in nature, it forms one of several necessary steps to inform the future development of system-wide approaches to collecting, reporting and comparing surgical outcomes in Canada.

## Methods

We conducted a secondary analysis of a sample of consecutive patients scheduled for select elective inpatient or outpatient surgery in six acute hospitals in the geographically defined Vancouver Coastal Health (VCH) authority, home to over 1.25 million residents. Patients were eligible to be included if they were scheduled for an elective surgery that was included in this study, at least 18 years of age, able to communicate in English, community-dwelling and patients of surgeons who had agreed to have their patients participate. Patients were excluded if their surgery was to be performed two-weeks or less from time of scheduling to remove urgent cases.

All participants were contacted within two weeks of being registered for their surgery and preoperatively completed a survey that included the PRO. Participants were contacted by phone up to two times to complete the same survey six months postoperatively and when early recovery from their surgery was expected to have occurred.

The preoperative survey was collected from September 2015 and the postoperative survey was collected until September 2020. For orthopaedic surgery patients, the survey was initiated in September 2016.

### Case mix categorisation

Canada uses separate case mix algorithms for inpatient and outpatient treatment. The Case Mix Groups+ (CMG+) was applied to inpatient hospital discharges while outpatient discharges were case mix adjusted using the Comprehensive Ambulatory Classification System (CACS). CMG+ and CACS are analogous to diagnoses-related groups (DRGs), where patients are uniquely assigned to a category for hospital payment based on their surgery. The CMG+ and CACS algorithms are maintained by the Canadian Institute for Health Information and independent of this study.^16,17^

CMG+ case mix categories considered in this study included: Hysterectomy with Non-Malignant Diagnosis (Gynaecology), Reduction Gastroplasty Without Bypass (General Surgery), Endoscopic Large Intestine/Rectum Resection without Colostomy (Colorectal Surgery) and Major Foot Intervention except Soft Tissue without Infection (Orthopaedics). The inclusion of a cancer-related elective surgery sought to evaluate whether changes in health observed among colon cancer patients was similar to the changes reported by patients of non-cancer surgeries. CACS case mix categories included: Hernia Repair Open Approach (General Surgery), Plastic and Other Breast Intervention (Plastic Surgery), Sinus Intervention (Otolaryngology) and Cholecystectomy (General Surgery).

### PRO instrument

Participants completed the EQ-5D(5L) to measure their current health status.^18^ The EQ-5D(5L) has five items and one vertical visual analogue scale (VAS). Each item relates to a health domain: mobility, self-care, usual activities, pain/discomfort and anxiety/depression and is rated using one of five levels corresponding with the severity of problems the participant is experiencing in the domain. On the VAS, participants mark the scale indicating their overall health status. The scale ranges from 0 (i.e. ‘Worst imaginable health state’) to 100 (i.e. ‘Best imaginable health state’).

This study used two primary outcome measures: the EQ-5D(5L)’s utility values and VAS scores. Each participant’s pattern of item responses was first used to describe their health state preoperatively and postoperatively.^18,19^ Then, participant’s health state was matched with the EQ-5D(5L)’s utility values.^20^ The utility values were derived from a sample of Canadians and independent of this study.^21^ Utility values range from less than 0 (representing a health state considered worse than death) to 1 (representing a health state considered perfect health). The second outcome measure was participants’ VAS scores, describing their overall health status.

We collected PROs from participants in one of 122 CMG+ and CACS case mix categories. For brevity, we present the results from four inpatient and four outpatient case mix categories, which we selected based on the representation across surgical specialities, volume of PROs returned by participants and clinician feedback.

### Analyses

Patient participation was calculated by comparing eligible patients with the number of participants. Some participants did not complete and return their postoperative survey and the percentage of participants that did not complete their postoperative survey was calculated and reported. Participants’ PRO data were linked with their inpatient or outpatient hospital discharge summary to obtain their case mix category. From the hospital discharge data, participants’ age at surgery, sex and Charlson Comorbidity Index were calculated. The Charlson Comorbidity Index is an ordinal value that represents the gradient of patients’ morbidity burden.^22^

All analyses were presented by case mix categories, reporting results by CMG+ or CACS category. Mean and standard deviations of preoperative and postoperative values of the EQ-5D(5L) utility value and VAS score were calculated. Paired t-tests were used to measure the statistical significance of the mean change in preoperative and postoperative values for each CMG+ or CACS category.

For each CMG+ or CACS category, the distributions of participants’ preoperative and postoperative EQ-5D(5L) utility values and VAS scores were calculated. All statistical analyses used Statistical Analysis Software (SAS, version 9.4).

### Ethics approval

The University of British Columbia’s Research Ethics Board approved this study (H11-02179).

## Results

Among eligible patients, the overall preoperative participation rate was 44.8% and the postoperative follow-up was 72.0% of participants. Participation varied among inpatient CMG+ and outpatient CACS case mix categories, ranging from 40.7% among otolaryngology patients (Sinus Intervention; *n* = 563) to 47.3% among orthopaedic patients (Major Foot Intervention except Soft Tissue without Infection; *n* = 244).

Table 1 describes the characteristics of participants within the case mix categories. Outpatient surgeries were more common than inpatient surgeries, and there were more female participants than males, consistent with the case mix groups selected, such as hysterectomy for benign conditions and delayed breast reconstruction. Other than among participants having surgery for colorectal cancer (case mix group Endoscopic Large Intestine/Rectum Resection without Colostomy), the comorbidity burden was very low and elective surgeries in these hospitals were predominantly conducted on participants with no other illnesses reported on their hospital discharge summary.

**Table 1.** Demographic and clinical characteristics of patients in inpatient and outpatient case mix categories.

Case mix group	Count	Mean (SD) age	Percent female	Mean (SD) Charlson
*Inpatient CMG+*				
Hysterectomy with Non-malignant diagnosis	261	55.5 (11.7)	100.0	0.05 (0.27)
Reduction gastroplasty without bypass	190	48.7 (10.2)	85.3	0.34 (0.59)
Endoscopic large intestine/Rectum resection without colostomy	212	65.4 (12.1)	50.0	1.96 (2.38)
Major foot intervention except soft tissue without infection	87	60.7 (10.2)	62.1	0.18 (0.42)
*Outpatient CACS*				
Hernia repair, open approach	309	65.1 (11.9)	10.1	0.05 (0.22)
Plastic and other breast intervention	267	53.5 (12.2)	98.9	0.18 (1.02)
Sinus intervention	197	55.8 (13.0)	45.2	0.05 (0.28)
Cholecystectomy	142	57.3 (12.1)	77.5	0.06 (0.27)

**Figure 1.**
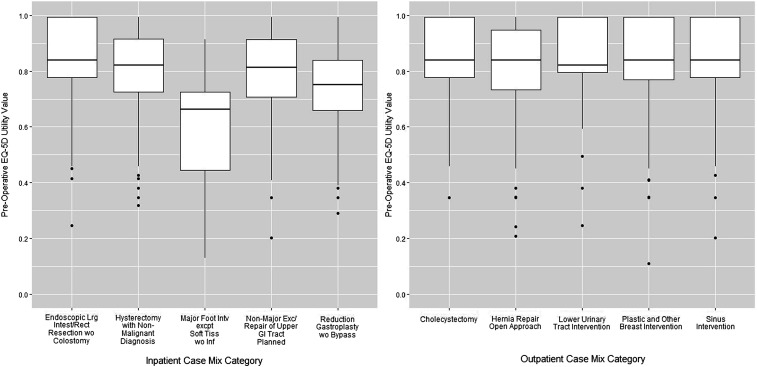
Boxplot of preoperative EQ-5D utility values, presented for inpatient and outpatient case mix categories.

Figure 1 illustrates the distribution of preoperative EQ-5D(5L) utility scores for all case mix categories. The figure highlights that preoperative utility values tended to be lower among participants scheduled for inpatient surgery compared with outpatient surgery. The CMG for Major Foot Intervention except Soft Tissue without Infection represented participants that reported the lowest preoperative health status. Detailed statistics for each case mix category are provided in the Online Supplement.

Table 2 presents EQ-5D utility values and VAS at preoperative and postoperative time points. There was a statistically significant improvement in both mean EQ-5D utility and VAS values in each of the eight case mix categories. The largest gain in mean EQ-5D utility and VAS values was among participants in the Reduction Gastroplasty Without Bypass case mix category.

**Table 2.** EQ-5D utility values and VAS, shown for preoperative and postoperative time points.

Case mix group	Count	EQ-5D utility value			EQ-5D VAS		
		Mean (SD) preoperative	Mean (SD) postoperative	T-test *p*-value	Mean (SD) preoperative	Mean (SD) postoperative	T-test *p*-value
*Inpatient CMG+*							
Hysterectomy with Non-malignant diagnosis	261	0.8120 (0.15)	0.9092 (0.12)	<0.01	76.0 (16.3)	82.7 (12.6)	<0.01
Reduction gastroplasty without bypass	190	0.7367 (0.17)	0.8882 (0.13)	<0.01	60.6 (15.9)	81.1 (11.6)	<0.01
Endoscopic large intestine/Rectum resection without colostomy	212	0.8610 (0.14)	0.9079 (0.13)	<0.01	77.8 (16.9)	81.8 (14.0)	<0.01
Major foot intervention except soft tissue without infection	87	0.6103 (0.17)	0.7176 (0.17)	<0.01	63.5 (17.8)	70.3 (16.9)	<0.01
*Outpatient CACS*							
Hernia repair, open approach	309	0.8180 (0.15)	0.8981 (0.14)	<0.01	79.4 (14.3)	83.6 (14.0)	<0.01
Plastic and other breast intervention	267	0.8438 (0.15)	0.8705 (0.17)	0.01	79.0 (14.8)	81.8 (15.5)	<0.01
Sinus intervention	197	0.8389 (0.14)	0.9020 (0.16)	<0.01	73.6 (17.7)	80.9 (14.9)	<0.01
Cholecystectomy	142	0.8529 (0.12)	0.9321 (0.12)	<0.01	76.7 (12.9)	83.5 (11.8)	<0.01

**Figure 2.**
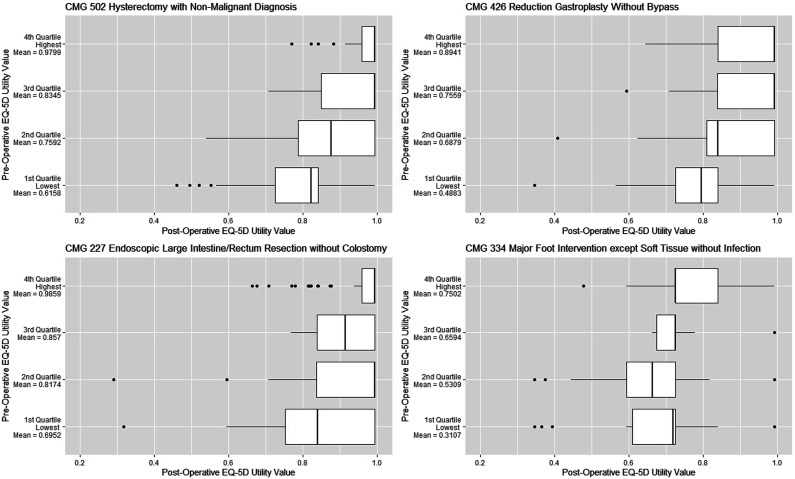
Boxplots of participants’ postoperative EQ-5D(5L) utility values, presented for four inpatient CMG+ case mix categories. Participants were stratified into four quartiles based on their preoperative utility values.

Participants with the lowest preoperative utility values tended to experience the largest gain in health although they were unlikely to reach the highest utility values experienced by participants with the highest preoperative utility values (Figure 2). Participants with the highest preoperative utility values experienced the smallest gains.

**Figure 3.**
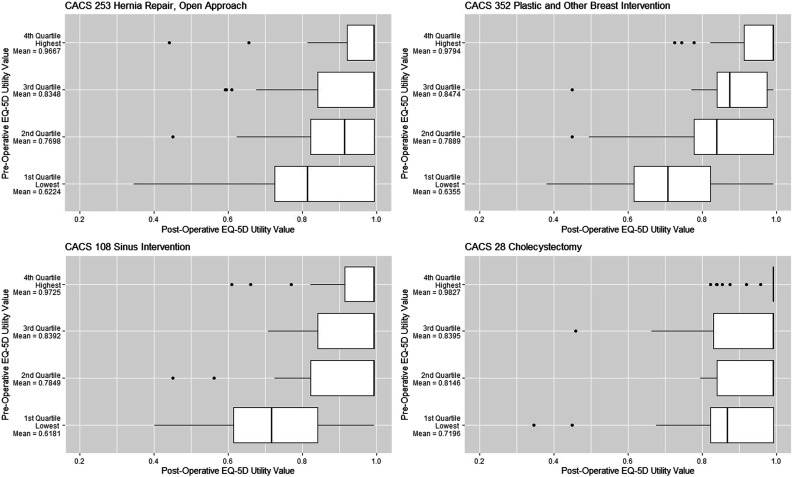
Boxplots of participants’ postoperative EQ-5D(5L) utility values, presented for four outpatient CMG+ case mix categories. Participants were stratified into four quartiles based on their preoperative utility values.

Similarly, among outpatient case mix categories, participants with the lowest preoperative utility values tended to experience the largest gain in utility values (Figure 3). Participants with the lowest preoperative utility values also tended to have experienced larger variability in their postoperative utility values compared with participants with higher preoperative utility values.

## Discussion

This was an exploratory study that was based on a standardised measure of health status and included a range of case mix categories. The literature has not been clear whether gains in health would have been expected to be similar within or between surgical case mix categories. Although presentation of the results was complex owing to the multidimensionality of participants’ utility and VAS values, each inpatient and outpatient case mix category was associated with a statistically significant gain in health status (*p* < .01 or lower) as measured by the utility value and VAS.

While we did not expect to observe similar gains in health between case mix categories a priori, the results highlighted some unambiguous findings. First, participants with the lowest health status tended to experience the largest gains, with bariatric surgery tending to have gained more than other case mix categories. Second, the healthiest patients in each case mix category reported very small gains in health status. These findings are consistent with other research identifying factors associated with variation in PROs^11,23^ and research showing a relationship between PROs and length of stay.^24^

This study included one cancer-related case mix category for comparative purposes. Because of limitations in the data, it was unclear whether this surgery occurred because the patient’s cancer was identified during screening rather than through presentation of symptoms. This raises two potential issues regarding the application of the EQ-5D for measuring the impact of surgery among cancer patients. First, the EQ-5D may not be sufficiently sensitive to measure changes in health status among cancer surgery patients who have experienced few or no symptoms, such as colon or prostate cancer. In these cases, other PROMs may be better suited for measuring mental health-related effects, such as symptoms of depression (PHQ-9) or anxiety (GAD-7). Second, since the treatment of colon cancer spans a number of treatment modalities, measuring change in health may need to incorporate a broader episode of cancer treatment, not just the surgical encounter.

This study highlights cautions pertaining to measuring and comparing case mix-based surgical outputs with PROs in Canada. First, completing PROs was not compulsory and fewer than one-half of patients completed their PROs. Higher levels of participation are recommended to minimise the risk to the validity of the data^23,25^ and maximise credibility of the findings. Second, although the EQ-5D(5L) is widely used, the instrument may not be sufficiently sensitive to detect meaningful changes in patients’ symptoms or function outcomes for all surgeries, suggesting that the instrument could be paired with an appropriate condition-specific instrument. Future work should focus on increasing participation and including condition-specific PROMs; also, further research is needed to assess the role of contextual variables on operative outcomes including wider determinants of health.

This study had additional limitations. After discharge, participants’ postoperative care, privately paid drugs, medical devices or therapies, were unobservable. Therefore, some portion of patient-level differences in their health status between and within CMG+ or CACS categories may be attributable to unmeasured interventions (or lack thereof) provided in the community.

## Conclusion

This study demonstrated that it was feasible to collect and report PROs for inpatient and outpatient surgeries for a range of case mix categories across a system of hospitals applying a standard protocol. While the study found that gains in health status occurred in all case mix categories, the magnitude of gains varied by case mix category and preoperative health status. The largest gains were experienced by those with the lowest preoperative health status.

## Supplemental Material

Supplemental Material Case mix-based changes in health status: A prospective study of elective surgery patients in Vancouver, Canada Click here for additional data file. Supplemental Material for Case mix-based changes in health status: A prospective study of elective surgery patients in Vancouver, Canada by Jason M Sutherland, R Trafford Crump, Ahmer A Karimuddin, Guiping Liu, Kevin Wing, Arif Janjua and Kathryn Isaac in Journal of Health Services Research & Policy
