# Supplementary material for: Case mix-based changes in health status: A prospective study of elective surgery patients in Vancouver, Canada
Source: J Health Serv Res Policy. 2023 Jun 11;28(4):215–21. doi: 10.1177/13558196231182630 (PMC10515465; doi:10.1177/13558196231182630)
Supplement: Supplemental Material Case mix-based changes in health status: A prospective study of elective surgery patients in Vancouver, Canada [file sj-pdf-1-hsr-10.1177_13558196231182630.pdf]

Online Supplement

Journal of Health Services Research & Policy
Comparing patient-reported outcomes of inpatient and outpatient elective surgeries through hospitals' case mix categories
Sutherland, Crump, Karimuddin, et al.

Preoperative EQ-5D utility values and VAS. Higher values are associated with better health.

	Preoperative EQ-5D utility values				Preoperative VAS values			
Case mix group	Minimum	Median	Mean	Maximum	Minimum	Median	Mean	Maximum
Inpatient CMG+								
Hysterectomy with Non Malignant Diagnosis	0.3110	0.8260	0.8120	1	29.0	80.0	76.0	100
Reduction Gastroplasty Without Bypass	0.2830	0.7360	0.7367	1	15.0	62.0	60.6	100
Endoscopic Large Intestine/ Rectum Resection without Colostomy	0.2390	0.8260	0.8610	1	10.0	80.0	77.8	100
Major Foot Intervention except Soft Tissue without Infection	0.1230	0.6640	0.6103	0.844	20.0	65.0	63.5	90
Outpatient CACS								
Hernia Repair, Open Approach	0.1990	0.8430	0.8180	1	20.0	80.0	79.4	100
Plastic and Other Breast Intervention	0.3400	0.8260	0.8438	1	30.0	80.0	79.0	100
Sinus Intervention	0.3400	0.8440	0.8389	1	20.0	75.0	73.6	100
Cholecystectomy	0.3400	0.8440	0.8529	1	28.0	80.0	76.7	100
